# *BRCA1* and *BRCA2* Mutations Other Than the Founder Alleles Among Ashkenazi Jewish in the Population of Argentina

**DOI:** 10.3389/fonc.2018.00323

**Published:** 2018-08-21

**Authors:** Angela R. Solano, Natalia C. Liria, Fernanda S. Jalil, Daniela M. Faggionato, Pablo G. Mele, Alejandra Mampel, Florencia C. Cardoso, Ernesto J. Podesta

**Affiliations:** ^1^Genotipificación y Cáncer Hereditario, Centro de Educación Médica e Investigaciones Clínicas “Norberto Quirno” (CEMIC), Ciudad Autónoma de Buenos Aires, Buenos Aires, Argentina; ^2^Facultad de Medicina, Instituto de Investigaciones Biomédicas (INBIOMED), Universidad de Buenos Aires-CONICET, Ciudad Autónoma de Buenos Aires, Buenos Aires, Argentina; ^3^Hospital Universitario, Instituto de Genética, Universidad Nacional de Cuyo, Mendoza, Argentina

**Keywords:** non-founder Ashkenazi *BRCA1/2* mutations, Ashkenazi Jewish, hereditary breast and ovary cancer, *BRCA1*, *BRCA2*

## Abstract

In Ashkenazi Jewish (AJ) high risk families 3 mutations [2 in *BRCA1* (c. 68_69del and c.5266dup) and 1 in *BRCA2* (c.5946del)] account for the majority of high risk breast and ovarian cancer cases in that ethnic group. Few studies with limited number of genotyped individuals have expanded the spectrum of mutations in both *BRCA* genes beyond the 3 mutation panel. In this study, 279 high risk individual AJ were counseled at CEMIC (Centro de Educación Médica e Investigaciones Clínicas), and were genotyped first for the 3 recurrent mutation panel followed by Next Generation Sequencing (NGS) of *BRCA1 BRCA2* in 76 individuals who tested negative for the first genotyping step. Of 279 probands (259 women), 55 (50 women) harbored one of the 3 mutations (19.7%); Of 76 fully sequenced cases (73 women), 6 (5 women) (7.9%) carried a pathogenic mutation: in *BRCA1*, c.2728C>T - p.(Gln910^*^); c.5407-?_(^*^1_?)del and c.5445G>A - p.(Trp1815^*^); in *BRCA2*, c.5351dup - p.(Asn1784Lysfs^*^3); c.7308del - p.(Asn2436Lysfs^*^33) and c.9026_9030del - p.(Tyr3009Serfs^*^7). Of 61 mutation carriers the distribution was as follows: 11 cancer free at the time of genotyping, 34 female breast cancer cases with age range 28–72 years (41.6 ± 9.3), 3 male breast cancer cases with age range 59–75 years (65 ± 7.3), 6 breast and ovarian cancer cases with age range 35–60 years (breast 40.4 ± 5.2; ovary 47.8 ± 7.2) and 7 ovarian cancer cases with age range 41–77 years (60.6 ± 13.3). This information proved highly useful for counseling, treatment, and prevention for the patient and the family. In conclusion comprehensive *BRCA1/2* testing in AJ high risk breast ovarian cancer cases adds valuable clinically relevant information in a subset of cases estimated up to 7% and is therefore recommended.

## Introduction

Inherited pathogenic mutations in *BRCA1* (OMIM^*^113705) or *BRCA2* (OMIM^*^600185) substantially increase lifetime risk for breast, ovarian and to a lesser extent other cancer types defining individuals who carry *BRCA1* or *BRCA2* cancer predisposing mutations is valuable for both cancer cases and unaffected family members: targeted treatment in the form of PARP inhibitors is available for mutation carrying patients (1, 2), and early detection schemes and risk-reducing strategies are offered to asymptomatic mutation carriers (3, 4).

Near 300 *BRCA1* and *BRCA2* pathogenic mutations in the Argentine population have been reported by our group (5, 6) routinely deposited in Leiden Open Variation Database 3.0 (7) and Leiden Open Variation Database–Chapter for Argentina (8), most previously described in other world populations with ~10% of novel pathogenic variants.

The estimated frequency of pathogenic germline mutations in *BRCA1* and *BRCA2* genes in the general population in several outbred populations vary between 1:300 and 1:800, respectively (4). However, in inbred populations, with AJ as one of the most frequently studied examples, the spectrum of *BRCA* mutations is limited with higher rates in the general population. In this ethnic group 1/40 individuals is a carrier one of 3 recurrent mutations in *BRCA1* [185delAG: c.68_69del (rs386833395) and 5382insC: c.5266dup (rs80357906)], and *BRCA2* [6174delT: c.5946del (rs80359550)]. Such high rates in the general population and consecutive breast and ovarian cancer cases enabled effective use of cancer genetics services for AJ women (9).

For high risk AJ individuals not carrying one of these 3 founder alleles, the probability for other pathogenic mutations in *BRCA1* or *BRCA2* has rarely been reported (10–13). In the current study we report on an extended analysis of *BRCA1/2* by comprehensive next generation sequencing and analysis of point mutations and large rearrangements in Ashkenazi patients with personal and/or family history of cancer qualifying for the panel analysis and testing normal for the 3 founder mutations.

## Materials and methods

### Study subjects

The study focused on Ashkenazi Jewish individuals recruited from those referred for counseling and genotyping at CEMIC (Centro de Educación Médica e Investigaciones Clínicas) between 2009 and 2017. Eligibility criteria for patient selection are based on the NCCN guidelines [National Comprehensive Cancer Network. Genetic/Familial High-Risk Assessment: Breast and Ovarian (Version 1.2018) https://www.nccn.org/ Accessed March 30, 2018]. For individuals of AJ origin, with no known familial mutation, were first tested for the 3 AJ specific mutations (see below genotyping methodology). Then, for high risk individuals of AJ who tested negative for the three mutations *BRCA* comprehensive *BRCA* genetic testing was carried out (see below genotyping platform). Study eligibility after genetic counseling required signing an informed consent as part of the routine procedures for genetic analysis (including Ethics Committee approval) at CEMIC, which also complies with the Traditional Pretest Counseling for Susceptibility Testing (purpose of testing) described in the American Society of Clinical Oncology Policy Statement Update (14).

### *BRCA1/2* testing platforms

Genomic DNA of the 279 blood samples was isolated by MagNA Pure® LC instrument with total DNA isolation kit I (Roche Diagnostics). All samples were analyzed by Sanger sequencing of PCR amplified fragments for the 3 founder Ashkenazi mutations c.68_69del and c.5266dup in *BRCA1* and c.5946del in *BRCA2*.

Analysis of comprehensive *BRCA1/2* sequencing and large rearrangements by Multiple Ligation-dependent Probe Amplification assay (MLPA) for eligible individuals was performed by Next Generation Sequencing (NGS) by using the Ion AmpliSeq*BRCA1/2* community panel, as it allows to amplify the entire coding sequences of *BRCA1* and *BRCA2*, including 20–50 bases of adjacent intronic sequence of each exon. The assay is designed to ensure at least 200X total coverage/base. Sequencing of the amplified regions was performed with the next generation platform Personal Genome Machine® System, as previously described (6). Rare coding sequences with low coverage were analyzed by Sanger sequencing to ensure higher coverage rates. The raw signal data and the sequence reads were processed with Ion Torrent Suite software (Thermo Fisher Scientific) on a Torrent server. After data analysis, single nucleotide variants, insertions, deletions, and splice site alterations were registered, and all variants detected were reported. Sanger sequencing was used to confirm all clinically relevant variants detected (class 3, 4, and 5). Clinical significance was determined according to the reference databases: ClinVar (15), LOVD3.0 (7), and UMD (16) as of March 2018.

For missense mutations not reported or reported with uncertain clinical significance (VUS), *in silico* programs were used to predict the change in protein function using software Align-GVGD (http://agvgd.iarc.fr/), SIFT (http://sift.bii.a-star.edu.sg), and Mutations Taster (http://www.mutationtaster.org/).

Large rearrangements were measured by MLPA using SALSA MLPA Probemix P002 and P045 provided by MRC-Holland, and Coffalyser.net software was used for data analysis; we confirm the positive results with P087 and P077 for *BRCA1* and *BRCA2*, respectively.

## Results

### Participant's characteristics

**Figure 1:**
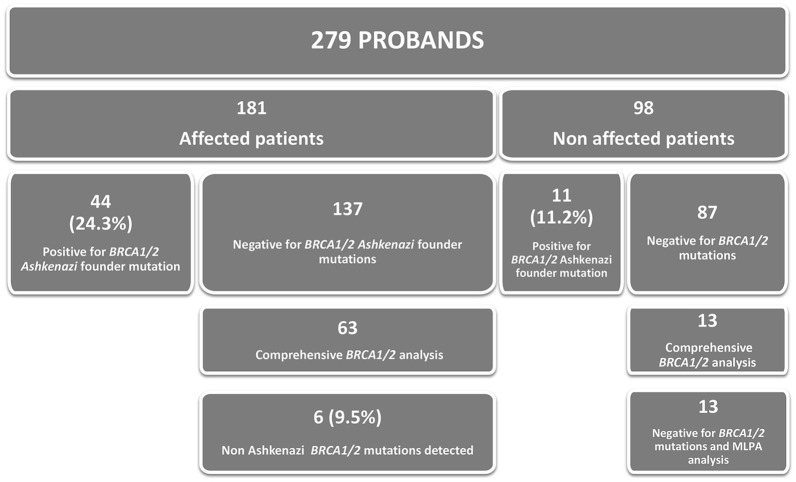
Patients of Ashkenazi origin with personal and/or family history (279 probands) were tested for the panel AJ founder mutations; among affected patients 24.3% were detected with a mutation while this rate was 11.2% in the non-affected individuals. Sixty three patients without AJ mutation were subjected to the comprehensive *BRCA1/2* analysis and 6 were diagnosed with a pathogenic mutation (9.5% from the analyzed probands). On the other hand, 98 were healthy individuals with family history of cancers related to *BRCA1/2* and, among them. The non-Ashkenazi mutations were detected always in affected patients.

Overall, we include 279 patients (Figure 1) recruited among 2009–2017 as depicted in Table 1. Age range at counseling and genotyping was 20–87 years; 181 (174 females) had cancer diagnoses (mean age 48.3 ± 11.2 years) and 98 were healthy, cancer free high risk individuals (mean age at genotyping 47.7 ± 11.8). Of cancer cases the distribution was as follows: of 174 cancer affected women, breast, 145 (age range 28–74 years, mean 47.0 ± 9.6), breast and ovarian 7 (age range breast 35–64 years, mean 44.3 ± 9.8; age range ovary 41–64 years, mean 49.2 ± 8.9), ovarian 19 (age range 18–78 years, mean 52.5 ± 16.6) and one each with pancreas (68 years), endometrium (58 years) and melanoma (26 years). For the 7 cancer affected men, 6 had breast cancer (age range 59–75 years; mean 65.5 ± 5.7) and 1 with prostate cancer (61 years).

**Table 1:** Analysis of the study subjects.

**Patients analyzed (Total = 279)**	**Patients with mutation (*n*)**	**Age range**	**Age mean ± *SD***
Healthy women	9	40–58	47.7 ± 5.3
Healthy men	2	47 & 76	61.5 ± 20.5
Breast cancer women	34	28–72	41.6 ± 9.3
Breast cancer men	3	59–75	65 ± 7.3
Ovary cancer	7	41–77	60.6 ± 13.3
Breast and ovary	6		
Breast		35–46	40.4 ± 5.2
Ovary		41–60	47.8 ± 7.2
Total with a mutation	61 (27.6%)^*^		

* *AJ mutations 55/279 (19.7%); Non-AJ mutations 6/76 (7.9%)*.

**Figure 2:**
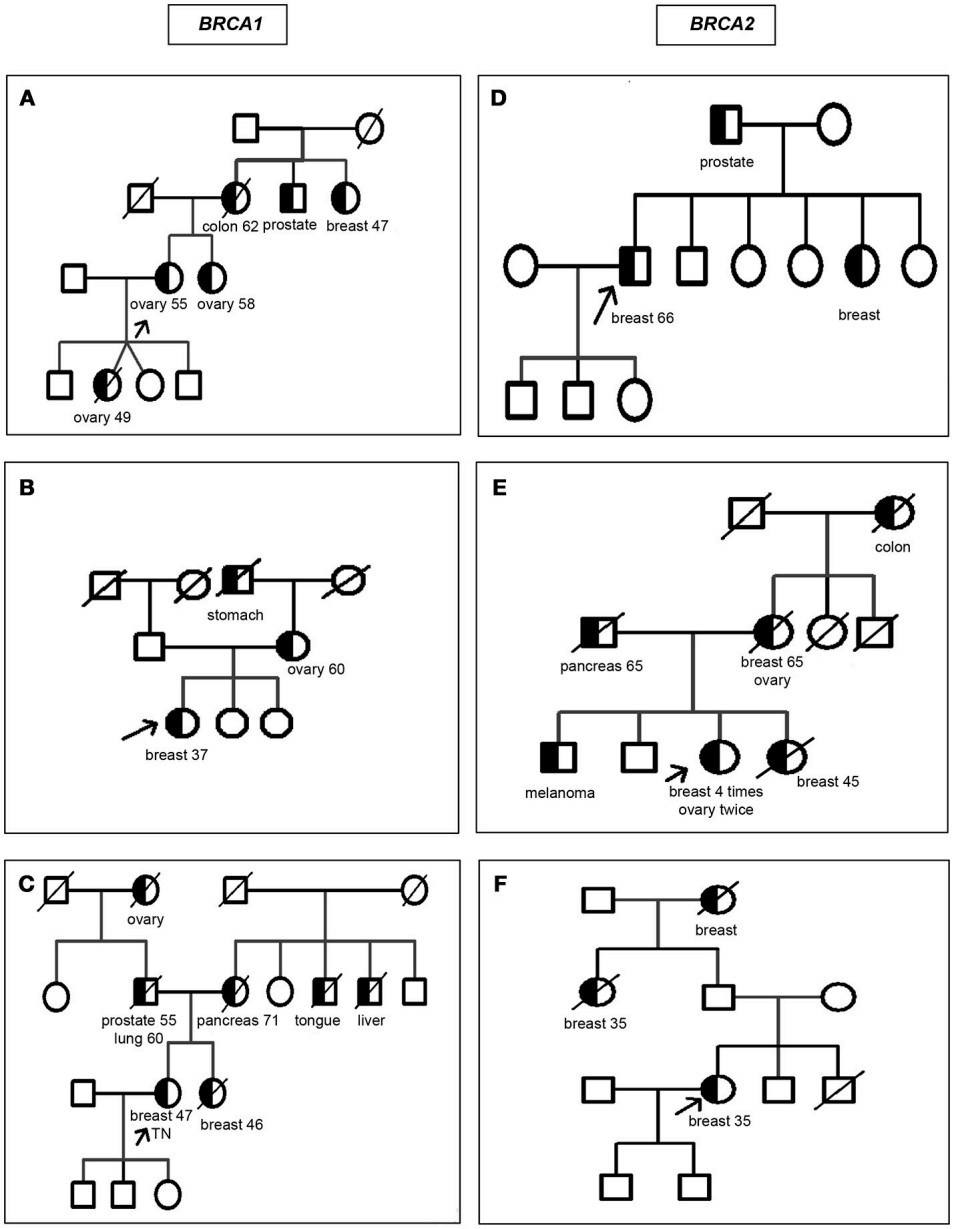
Pedigrees of the families carrying mutations in *BRCA1/2* other than the Ashkenazi founder mutations. Circles: women; squares: men; half blackened symbols: individuals affected with cancer; white symbols: unaffected individuals; TN: triple negative breast cancer; slash diagonal line: deceased. The cancer and age at diagnosis are indicated below each individual when available. An arrow indicates the proband. **(A)** *BRCA1* c.2728C>T - p.(Gln910*); **(B)** *BRCA1* c.5407-?_(*1_?)del; **(C)** *BRCA1*: c.5445G>A-p.(Trp1815*); **(D)** *BRCA2*: c.5351dup - p.(Asn1784Lysfs*3); **(E)** *BRCA2* c.7308del - p.(Asn2436Lysfs*33); **(F)** *BRCA2* c.9026_9030del - p.(Tyr3009Serfs*7).

The 279 selected patients were first analyzed through the AJ founder mutation and, among those who tested normal, 76 patients were analyzed by the *BRCA1/2* comprehensive study; among the 6 patients with a non AJ mutation detected all but 2 were of full AJ origin, one was mixed Ashkenazi non Ashkenazi and the other mixed Ashkenazi and non-Jewish. The ages ranged from 20 to 87 years old; 181 of them were affected with a mean age of 48.3 ± 11.2 and 98 of them were healthy with a mean age of 47.7 ± 11.8. Table 1 summarizes the age range and mean age ± SD for subjects with a mutation detected separated by diagnosis and gender. As eligible patients we included men or women selected by their AJ ethnicity, with two exceptions (Figure 2): proband [B: *BRCA1* c.5407-?_(^*^1_?)del] was half Sephardi, and proband [D: *BRCA2*: c.5351dup - p.(Asn1784Lysfs^*^3)] was half non-Jewish, although this side was not the side associated with inheritance of the syndrome.

Overall, 61/279 genotyped cases (21.8%) harbored a *BRCA1* mutation (*n* = 44) or a *BRCA2* mutation (*n* = 17). Of these mutations all but 3 in *BRCA1* and 3 in *BRCA2* were one of the predominant AJ mutations in both genes.

Table 1 summarizes age at genotyping and/or cancer diagnosis and type and gender of all mutation carriers and the specific unique non founder mutations.

Figure 1 summarizes the genotype analysis of 279 individuals with the DNA sequenced for the panel of the 3 founder Ashkenazi mutations and full sequence by NGS technique and MLPA.

Table 1 lists the patients with a mutation detected, to be remarked women with diagnosis of breast cancer were the youngest (range starts at 28 years), mean age non-statistically different from women with diagnosis of both, breast and ovary cancer.

In Tables 2, 3 are detailed the mutations detected in females and males respectively.

**Table 2:** Analysis of mutations detected in female individuals.

**Female patients mutation analysis**	***n***	**%**	***n* (tumor type, age range or age@diag)**	**Healthy**
**Recurring Ashkenazi mutations**				
Total	259			
Gene: Mutation	50	19.3		9
*BRCA1*: c.68_69del -p.(Glu23Valfs^*^17)	24	9.3	18, Br (30-72); 1 Br/Ov (46, 47); 2, Ov (59, 77)	3 (^*^)
*BRCA1*: c.5266dup -p.(Gln1756Profs^*^74)	15	5.8	8, Br (28-46); 4, Ov (41-72); 2, Br/Ov (35,41; 37,45)	1
*BRCA2*: c.5946del -p.(Ser1982Argfs^*^22)	11	4.2	4, Br (30-56); 1 Br & Fallopian tube (64); 1 Br/Ov (46)	5
**Unique mutations (NGS)**				
Total	73			
Gene: Mutation	5	6.8		
*BRCA1*: c.2728C>T - p.(Gln910^*^)	1		Ov (55)	0
*BRCA1*: *c*.5407-?_(^*^1_?)del	1		Br (37)	0
*BRCA1*: c.5445G>A - p.(Trp1815^*^)	1		Br (47)	
*BRCA2*: c.7308del - p.(Asn2436Lysfs^*^33)	1		Br (38,45,47,52) & Ov (60, 64)	0
*BRCA2*: c.9026_9030del - p.(Tyr3009Serfs^*^7)	1		Br (35)	0

*n, number of probands; age@diag, age at diagnosis; Br, Breast Cancer; Ov, Ovarian Cancer*.

(^*^) a patient was carrier of the mutation in the gene MSH2: c.1906G>C - p.(Ala636Pro), also of Ashkenazi origin (17).

**Table 3:** Analysis of mutations detected in male individuals.

**Male patients mutation analysis**	***n***	**(%)**	**age@diag**	**Healthy**
**Ashkenazi mutation**				
Total	20			
A mutation found	5	25.0		2
*BRCA1*: c.68_69del -p.(Glu23Valfs^*^17)	2		Br (75)	1
*BRCA1*: c.5266dup -p.(Gln1756Profs^*^74)	0		0	0
*BRCA2*: c.5946del - p.(Ser1982Argfs^*^22)	3		Br (60); Br (59)	1
**Non-Ashkenazi mutation (NGS):**		0.0		0
Total	3			
A mutation found	1	33.3		
*BRCA2*: c.5351dup - p.(Asn1784Lysfs^*^3)	1		Br (66)	0

*n, number of probands; age@diag, age at diagnosis; Br, Breast Cancer*.

Figure 2 depicts the pedigrees for the six families with a non AJ founder mutation. Of the 6 mutations found, *BRCA2* c.7308del - p.(Asn2436Lysfs^*^33) has not been previously reported and is therefore novel while the other 5 have been reported in non-Jewish populations.

Regarding the families with a non-Ashkenazi mutation, the particular details of the family history of cancers related to *BRCA* was strong for the 6 probands, as shown in the pedigrees drawn in Figure 2. The description of the mutations are in Table 4, as follows: *BRCA1*: c.2728C>T - p.(Gln910^*^), rs397509004, c.5407-?_(^*^1_?)del, c.5445G>A - p.(Trp1815^*^), rs397509284 and *BRCA2*: c.5351dup - p.(Asn1784Lysfs^*^3), rs80359508, c.7308del - p.(Asn2436Lysfs^*^33), c.9026_9030del - p.(Tyr3009Serfs^*^7), rs80359741.

**Table 4:** Families with a non-AJ founder mutation in *BRCA1/2* from pedigrees drawn in Figure 2.

**Mutation (HGVS nomenclature)**	**Description**
*BRCA1*: c.2728C>T - p.(Gln910*) rs397509004	This is a rare mutation reported in ClinVar and we deposited in LOVD (Genomic Variant #0000206572). The proband with age 71 at testing had ovarian cancer at 55 and her daughter died from an ovarian cancer at 49. There is no information of the twin. The sister, also affected, tested positive for the mutation.
*BRCA1*: c.5407-?_(*1_?)del	This large rearrangement has been described many times in the databases. As shown in Figure 2, this half Sephardi patient had family history of stomach and ovary cancer.
*BRCA1*: c.5445G>A - p.(Trp1815^*^) rs397509284	This mutation is deposited by different laboratories at least 5 times at the LOVD. The proband had triple negative breast cancer (the type of cancer most frequently associated with *BRCA1* mutations) at 47, was tested at 49 and had a sister who died of breast cancer at 46.
*BRCA2*: c.5351dup - p.(Asn1784Lysfs^*^3) rs80359508	We have detected this mutation 10 times and it is reported in LOVD at least 49 times (including 7 from our laboratory) (18). The family history is relatively scarce in information, with a sister with breast cancer and the father with prostate cancer. The daughter and one of the sons tested normal for the mutation. Considering the origin of this mutation it may be the case that the cancer diagnosis from the Jewish side could be phenocopies, and for this reason the prevalence for *BRCA2* non AJ was also calculated non including this case.
*BRCA2*: c.7308del - p.(Asn2436Lysfs^*^33)	Novel, not reported in the databases and we deposited at LOVD (Genomic Variant #0000206927). The proband, tested at 67, has history of breast cancer 4 times, starting at 38, 45, 47, and 52 years of age; she also developed ovary cancer at 60 and 64. Two relatives were tested, a niece of 38 was found to be a non-carrier and a nephew at 41 an unaffected carrier.
*BRCA2*: c.9026_9030del - p.(Tyr3009Serfs^*^7), rs80359741	This mutation was detected 3 times in our laboratory and at least 10 more times deposited at LOVD. This mutation was associated to Spanish ascendancy (19).

*The following variant of uncertain significance (VUS) was detected in the samples analyzed by NGS: BRCA2: c.7232A>C - p.(Lys2411Thr), rs80358950 (we deposited at LOVD Genomic Variant #0000206714)*.

## Discussion

The likelihood that AJ high risk individuals who do not carry any of the predominant AJ mutations in *BRCA1* and *BRCA2* would harbor a unique *BRCA1* or *BRCA2* mutation in the present study was 7.9%. As the family D was self-reported as half-non AJ, the resulting prevalence for *BRCA1/2* non AJ mutation excluding this family results to be 5 out of 76 cases (6.6%). This rate is line with previous studies, although there are only a few that have reported such a focused analysis (10–13). In those previous studies the rates were 4-5% of fully genotyped AJ cases. The rationale behind our two step sample analysis approach lies in the fact that non-routine tests in the Argentinian health care system requires approval by a specialized committee that tends to not approve oncogenetic testing for unaffected individuals despite having a significant family history of cancer.

A rather difficult task is to express the results of this study, as not all the patients without an Ashkenazi mutation were analyzed by NGS and MLPA. As a consequence, the percentages of mutations cannot be straightforwardly interpreted, which may constitute a limitation for expressing the current findings. However, our results still remain valid, as even if all samples normal for the Ashkenazi panel had been tested for the comprehensive *BRCA1/2* study and no other mutation would had been detected, the percentage obtained would still have been 3.3% (6 out of 181 affected patients).

The population targeted in this study was selected on the basis of clinically having features of inherited cancer syndrome. Overall, non AJ mutations were detected in 6.8% of female cancer cases; 4.1% in female breast cancer cases and 2.7% in ovarian cancer (alone or with breast cancer). These rates are higher than those previously reported for Ashkenazi population, likely due to different criteria used for patient selection (10, 11). The highest mutation rates were found in cases of ovarian cancer including breast cancer diagnosed in the same patient, 50%, with similar rates to what was previously published by our group in a description of 940 patients (6).

Worth highlighting, 11 out of these 13 mutations were among the Ashkenazi panel and two detected by full analysis. The two probands found to carry a mutation in *BRCA1/2* support the application of precision medicine: patients benefit from being considered for poly–(adenosine diphosphate–ribose) polymerase (PARP) inhibitor therapy (1, 2), while their families still qualify for prevention measures as the first goal in these genetic studies.

Family history of cancer in first degree relatives as a major selection criterion for selecting cases to be genotyped carries an inherent limitation, as gender specific cancer (e.g., ovarian cancer) cannot be used in cases where the mutation arises on the paternal side yet, the value of incorporating second or third degree relatives on either parental side has its own merits. Moreover there are cases where the family history is distinct or the paternal from the maternal sides and both should be taken into account and guide the genetic testing platform. A case that exemplifies this point is the patient who co-harbored the *MSH2* c.1906G>C - p.(Ala636Pro) (17) and *BRCA1* c.68_69del - p.(Glu23Valfs^*^17). These findings are of the utmost importance, as surveillance of the unaffected proband will focus on both Lynch and hereditary breast-ovary cancer syndromes, while genetic counseling will be crucial for both paternal and maternal relatives.

The results of this study support the guidelines recommending genetic testing for the recurrent *BRCA* mutations in all AJ breast and ovarian cancer patients (NCCN Clinical Practice in Oncology, Version 1.2018). In addition, the value, albeit limited to <8% more mutations, of an extended comprehensive *BRCA* genotyping for high risk Ashkenazim who are negative for the 3 predominant mutations needs to be offered and discussed with the patients at genetic counseling, regardless of the cost. This is particularly important as having a *BRCA* mutation has therapeutic implications especially in ovarian cancer cases, who can benefit from PARP inhibitor treatment.

## Author contributions

AS, FC, and EP discussed the conception of the work and take responsibility for data integrity and the accuracy of data analysis. FJ, NL, AM, and DF collected and organized patient samples. PM, FJ, and DF analyzed the literature and evaluated statistics. All authors critically revised the manuscript for important intellectual content.

### Conflict of interest statement

The authors declare that the research was conducted in the absence of any commercial or financial relationships that could be construed as a potential conflict of interest.
